# Characteristics of dental care-seeking behavior and related sociodemographic factors in a middle-aged and elderly population in northeast China

**DOI:** 10.1186/s12903-015-0053-3

**Published:** 2015-06-13

**Authors:** Lu Liu, Ying Zhang, Wei Wu, Ruibo Cheng

**Affiliations:** Department of Preventive Dentistry, School of Stomatology, China Medical University, Shenyang, 110002 China; Department of Epidemiology, School of Public Health, China Medical University, Shenyang, 110001 China

**Keywords:** Dental care-seeking behavior, Northeast China, Sociodemographic factors

## Abstract

**Background:**

The etiologies of oral disease are generally progressive and cumulative, such that compared with younger individuals, middle-aged and elderly people are at greater risk of active dental caries and periodontal disease risk. They usually suffer from multiple oral diseases, and obstacles to their use of dental care services are numerous.

**Objectives:**

The objectives of this study were to investigate the characteristics of dental care-seeking behaviors and related sociodemographic factors in a middle-aged and elderly population in northeast China.

**Methods:**

This was a cross-sectional study of 1188 subjects, including 792 middle-aged (35–44 years-old) and 396 elderly (65–74 years-old) residents of northeast China. Information on dental care-seeking behaviors and sociodemographic characteristics was collected during face-to-face structured interviews conducted between May and June 2010. Chi square tests, Ridit scoring, and multivariate logistic regression analysis were employed to characterize dental care-seeking behaviors and their associations with sociodemographic factors.

**Results:**

A greater proportion of middle-aged participants reported a need for dental visits compared with the elderly participants (75.8 % vs. 60.9 %; *P* < 0.01), as did more urban that rural residents (*P* < 0.05). The majority of individuals in both the middle-aged and elderly groups obtained for dental care at their own expense, and they predominantly chose private dental clinics. Ridit analyses showed that education level and income were significantly associated with oral care in both middle-aged and elderly people (*P*s < 0.05). In addition, logistic regression analysis indicated that rural residence was negatively associated with dental visits in both middle-aged (odds ratio = 0.649, 95 % confidence interval: 0.447–0.884) and elderly (odds ratio = 0.604, 95 % confidence interval: 0.394–0.924) individuals.

**Conclusion:**

The rate of dental care visits is low in the middle-aged and elderly populations of northeast China. Among sociodemographic factors, education level and income are positively associated with dental care visits, and rural residence is negatively associated with the frequency of such visits.

## Background

The prevention of oral disease and the promotion of oral health have been established as high priorities by the World Health Organization [1]. The etiologies of oral disease are generally progressive and cumulative, such that compared with younger individuals, elderly people are at greater risk of active dental caries and periodontal disease risk [2–4]. However, although middle-aged and elderly people are aware of the importance of oral health to their quality of life [5], the majority of them seek dental care only when they experience toothaches [6]. As consequence, middle-aged and elderly individuals usually suffer from multiple oral diseases, and obstacles to their use of dental care services are numerous [7]. As the population ages and the retention rate of natural teeth increases, the demand for dental care becomes more urgent in middle-aged and elderly people than in adolescents [8].

In the traditional sense, obtainment of dental care services is related to the ability to access oral health resources [9]. If dental care-seeking behavior can be characterized in middle-aged and elderly people, then the reasons underlying the obstacles to timely and proper dental care might be identified. In addition to human resources such as dental care and the ability of the patient to pay, access to dental care is determined to a considerable extent by sociodemographic factors, including age, sex, education level, socioeconomic status, and residence category [10, 11], all of which impact overall [12, 13] and oral [14, 15] health. On a social level, these factors affect the availability of public oral health services, while at the family level, they impact income, parental education level, and oral health beliefs and habits. Moreover, at the level of the individual, sociodemographic factors affect use of public oral health services, as well as personal oral hygiene habits. Given their collective impact on oral health, disparities in sociodemographic factors, such as education level [16], income [17], social status [18], and location [19], are associated with the occurrence of oral health disorders [20, 21].

While the effect of sociodemographic factors on oral health and dental care-seeking behavior has been well characterized in developed countries [22–24], this is not the case in developing countries. Compared with developed countries, limitations in China in the number of oral health care institutions and the coverage of community oral health services combine to compromise access to oral health care services and increase the incidence of oral disease. In addition, health insurance coverage is higher in urban areas compared to rural areas in China. Furthermore, medical insurance is not nationalized, and coverage is mainly provided by employers. As a result, many patients have to pay for oral health care themselves.

China is characterized by striking disparities in sociodemographic factors among its seven geographic and economic mega-regions. In south China, for example, female sex, urban residence, education level, and income are positively associated with oral health care-seeking behavior [25, 26], while in southwest China [27], poorer oral health knowledge and economic status collectively restrict demand for oral health care in elderly individuals. Moreover, in central China, Zhou et al. [28] observed that education level and wearing of dentures were significantly associated with oral health. However, the relationships between sociodemographic factors and oral health-seeking behavior in middle-aged and elderly populations have yet to be characterized in northeast China, which is an important industrial base. Estimates of the prevalence of dental caries (>60 %) and periodontal health (<5 %) in middle-aged and elderly individuals by the third Chinese Oral Health Epidemiological Survey [29, 30] in 2005 indicate an urgent need for dental care in northeast China. We hypothesized that sociodemographic factors also affect dental care-seeking behavior in individuals in northeast China. The objectives of this study were to characterize the relationship between such factors and dental care-seeking behaviors in middle-aged and elderly populations in northeast China.

## Methods

### Study population

This was a cross-sectional study of 1188 subjects, including 792 middle-aged (35–44 years-old) and 396 elderly (65–74 years-old) residents of northeast China. Fieldwork was conducted between May and June 2010. The subjects were randomly selected from both urban (three cities, namely Shenyang, Jinzhou and Wafangdian) and rural areas (three counties, namely Liaozhong, Dengta, and Yixian) using multistage stratified random sampling. In the first stage, urban and rural areas were stratified. Urban areas were divided into three categories, namely, large, medium, and small, based on population size. The large city chosen for the study was the capital city. A medium city and a small city were randomly selected. Rural areas (counties) were divided into to three categories, namely, high, middle, and low, based on their economic status, and one county was randomly selected from each category. In the second stage, in each district of the selected cities, three streets were randomly selected, from each of which two communities were randomly selected. In each of the selected counties, three towns were randomly selected, from each of which two villages were randomly selected. To be eligible for inclusion, subjects were required to have been living in that area for more than 6 months prior to the start of the study. This study was approved by the Ethics Committee of School of Stomatology, China Medical University.

### Data collection and quality control

After obtaining written informed consent, a structured interview was conducted with each individual by two investigators, both registered dentists that received unified training prior to the study, to collect data on residence, sex, and nationality, as well as information on knowledge, attitudes, and habits of oral health, use of fluoride toothpaste, eating habits, and perceived need for and frequency of dental care visits. The questionnaire used in the present study is the standard questionnaire developed for the Third Chinese Oral Health Epidemiological Survey [31], with reliability and validity verified in Chinese people. Responses to the questionnaire were obtained and recorded by the investigators. Conditions were consistent between survey site, and the questionnaire was asked, recorded, and collected in a unified manner by the investigators. No clinical examinations were performed.

### Statistical analysis

Self-reported questionnaire data were entered into the database using Epidata 3.0, and analyzed with SPSS v13.0 (SPSS Inc., Chicago, IL, USA) in 2013. Chi square tests were conducted to compare the distributions of dental care visits and habits between the study populations. Ridit analysis was conducted for the ordered grouped data in the questionnaire, with the variables ranked in the manner of less to more frequent or less to more severe. Multivariable logistic regression analysis using odds ratios and 95 % confidence intervals was used to examine the associations between sociodemographic factors and dental care-seeking behaviors. Sociodemographic factors identified by univariate analyses were subjected to multivariable logistic regression analysis as independent variables. For dependent variables, positive dental care-seeking behavior (at least one dental care visit during the previous 2 years) was set as 1, while negative dental care-seeking behavior (no dental care visit during the previous 2 years) was set as 0. A *P* < 0.05 was considered as statistically significant.

## Results

### General information

The ratio of male to female participants in the study population was 1:1. A valid questionnaire was obtained from 100 % (1188/1188) of participants using face-to-face interviews. Among these, 17.7 % of middle-aged individuals and 19.7 % of elderly individuals had at least one dental care visit (*χ*^*2*^ = 0.719, *P* = 0.396). In the elderly group, the proportion of urban residents who reported a dental care visit was significantly higher than for rural residents (*P* = 0.002) (Table 1).

**Table 1:** Characteristics of dental care-seeking behavior in middle-aged and elderly people in northeast China, n (%)

Characteristic	Middle-aged					Elderly					P*	*χ* ^*2*^
	Urban area	Rural area	Total	*P*	*χ* ^*2*^	Urban area	Rural area	Total	*P*	*χ* ^*2*^		
Number of subjects, *n*	400	392	792			198	198	396				
Having dental care visit	78 (19.5)	62 15.8)	140 (17.7)	0.174	1.846	51 (25.8)	27 (13.6)	78 (19.7)	0.002	9.196	0.396	0.719
Is there a need for dental care visit?				0.034	6.759				0.049	6.047	<0.001	31.249
Yes	312 (78.0)	288 (73.5)	600 (75.8)			131 (66.2)	110 (55.6)	24 (60.9)				
No	76 (19.0)	100 (25.5)	176 (22.2)			63 (31.8)	86 (43.4)	149 (37.6)				
Unknown	10 (2.5)	4 (1.0)	14 (1.8)			4 (2.0)	2 (1.0)	6 (1.5)				
Do you need the following dental care?				0.092	4.774							
Extraction, filling, or inlay	332 (83.0)	227 (57.9)	559 (70.6)			–	–	–	–			
Periodontal treatment	62 (15.5)	58 (14.8)	120 (15.2)			–	–	–	–			
Preventive dental care	117 (29.3)	65 (16.6)	182 (23.0)			–	–	–	–			
Reason for seeking dental care				0.237	2.879				0.001	14.639	0.453	1.585
Regular oral health checkup	15 (3.8)	13 (3.3)	28 (3.5)			12 (6.1)	0 (0.0)	12 (3.0)				
Acute or chronic toothache	328 (82.0)	338 (86.2)	666 (84.1)			159 (80.3)	177 (89.4)	336 (84.8)				
Other reasons	56 (14.0)	40 (10.2)	96 (12.1)			23 (11.6)	15 (7.6)	38 (9.6)				
Received dental care				0.195	3.274				<0.001	37.980	0.545	1.214
Extraction, filling, or inlay	379 (94.8)	318 (81.1)	697 (88.0)			184 (92.9)	197 (99.5)	381 (96.2)				
Periodontal treatment	36 (9.0)	20 (5.1)	56 (7.1)			31 (15.7)	0 (0.0)	31 (7.8)				
Preventive dental care	10 (2.5)	13 (3.3)	23 (2.9)			8 (4.0)	0 (0.0)	8 (2.0)				
Reason for not visiting a dentist				<0.001	35.520				0.155	6.662	<0.001	29.766
No oral diseases	158 (39.5)	209 (53.3)	367 (46.3)			90 (45.5)	91 (46.0)	181 (45.7)				
Oral disease was not severe	124 (31.0)	173 (44.1)	297 (37.5)			78 (39.4)	101 (51.0)	179 (45.2)				
No time	50 (12.5)	67 (17.1)	117 (14.8)			11 (5.6)	16 (8.1)	27 (6.8)				
Economic issue	130 (32.5)	80 (20.4)	210 (26.5)			67 (33.8)	89 (44.9)	156 (39.4)				
No dentists nearby	9 (2.3)	33 (8.4)	42 (5.3)			3 (1.5)	13 (6.6)	16 (4.0)				
Payment				<0.001	72.737				<0.001	66.231	0.084	6.658
Imbursement	10 (2.5)	1 (0.3)	11 (1.4)			8 (4.0)	3 (1.5)	11 (2.8)				
Health insurance	80 (20.0)	10 (2.6)	90 (11.4)			58 (29.3)	2 (1.0)	60 (15.2)				
New social health insurance	0 (0.0)	4 (1.0)	4 (0.5)			1 (0.5)	0 (0.0)	1 (0.3)				
At one’s own expense	307 (76.8)	376 95.9)	683 (86.2)			133 (67.2)	192 (97.0)	325 (82.1)				
Type of clinic				<0.001	150.303				<0.001	65.365	<0.001	42.544
Dental specialized hospital	26 (6.5)	34 (8.7)	60 (7.6)			15 (7.6)	0 (0.0)	15 (3.8)				
Provincial general hospital	0 (0.0)	0 (0.0)	0 (0.0)			4 (2.0)	0 (0.0)	4 (1.0)				
Municipal/county general hospital	128 (32.0)	27 (6.9)	155 (19.6)			31 (15.7)	15 (7.6)	46 (11.6)				
Community health service centers	10 (2.5)	13 (3.3)	23 (2.9)			8 (4.0)	7 (3.5)	15 (3.8)				
Dental care research institutions	10 (2.5)	13 (3.3)	23 (2.9)			12 (6.1)	0 (0.0)	12 (3.0)				
Dental care outpatients	62 (15.5)	7 (1.8)	69 (8.7)			15 (7.6)	0 (0.0)	15 (3.8)				
Private clinics	164 (41.0)	298 (76.0)	462 (58.3)			113 (57.1)	176 (88.9)	289 (73.0)				

*35–44 years-old *vs* 65–74 years-old

### Characteristics of dental care-seeking behavior

#### Perceived need for dental care

A significantly greater proportion of middle-aged participants answered yes to “is there a need for dental care visit?” compared to the elderly group (*P* < 0.001) (Table 1). In addition, a greater proportion of individuals who lived in urban areas had a perceived need for dental care visits than those who lived in the rural areas, both in the middle-aged and elderly populations (both *P* < 0.05).

#### Dental care visits during the previous year

There were no significant differences with respect to reasons for dental care visit and percentage receiving dental care between the middle-aged and elderly groups (Table 1). However, the reasons for the dental care visit differed between rural and urban residents in the elderly group (*P* = 0.001), with only urban residents reporting regular oral check-up as the reason for dental visit. In addition, the percentage of urban and rural elderly participants receiving dental care differed (*P* < 0.001), such that whereas all rural participants underwent extraction or obtained fillings or inlays, a proportion of elderly urban residents received periodontal treatment or preventative care.

#### Reasons for absence of dental care visits during the previous year

The reasons selected by participants for not visiting a dentist differed among the populations (Table 1). The most common self-reported reason for absence of dental care visits was “no oral disease” in both middle-aged and elderly groups. However, greater proportions of middle-aged participants in rural areas cited a perceived absence of disease, disease that was not severe, or a lack of time as reasons not to visit dentists.

#### Differences in forms of payment for dental care visits

The method of payment for dental care visits described by the participants did not differ between the middle-aged and elderly groups (Table 1). However, payment methods differed between rural and urban residents for both middle-aged and elderly groups (both *P* < 0.001). Almost all the participants in rural areas (95.9–97.0 %) received dental care at their own expense, whereas a smaller proportion (67.2–76.8 %) of urban residents paid out-of-pocket.

#### Differences in choices of dental care clinics

The type of dental care clinic in which the participants received oral care differed significantly between the middle-aged and elderly groups and between rural and urban residents (all *P* < 0.001) (Table 1). Whereas elderly patients primarily went to private clinics, middle-aged patients were more likely to receive care at specialized or municipal/county hospitals. Almost all elderly rural participants sought care at private clinics, whereas middle-aged participants also received care at outpatient clinics and municipal/county hospitals.

### Associations between education and income with oral care

A Ridit analysis showed that the level of education is significantly associated with the frequency of tooth brushing (*P* < 0.001) (Table 2). Specifically, participants with higher levels of education reported brushing their teeth more frequently.

**Table 2:** Tooth brushing frequency according to education level, n

Education level	Never or almost never	1–3 times/month	1 time/week	2–6 times/week	1 time/day	≥2 times/day	*P* ^*^
Illiteracy	28	6	4	42	55	13	<0.001
Primary school	18	7	9	57	133	33	
Middle school	6	4	11	43	213	118	
High school or above	7	1	5	15	212	148	

^*^Ridit analysis: *χ* ^2^ = 234.527

In addition, income level was associated with how long it had been since the participant last received dental care (*P =* 0.007) (Table 3). A larger proportion of participants who categorized their income as low had never visited a dentist, whereas a larger proportion of those who reported their income as high had recently (within the past 2 years) visited a dentist.

**Table 3:** Time since last dental care visit according to income, n

Income	<6 months	6–12 months	12–24 months	>24 months	Never visited a dentist	*P* ^*^
Low	96	57	118	337	308	0.007
High	33	27	40	102	70	

^*^Ridit analysis: *u* = 2.681 (low = 0.4878; high = 0.5413)

### Residential differences in access to oral health information

Access to oral health information significantly differed between urban and rural residents (*P* < 0.001) (Table 4). Individuals in urban areas had greater access, with more methods used to obtain information. A greater proportion of participants from rural areas (53.3 %; 315/590) reported having no access to oral health information compared to those in urban areas (39.80 %; 238/598).

**Table 4:** Number of methods used to obtain oral health information according to residence, n

Residence category	0	1	2	3	4	5+	*P* ^*^
Urban	238	114	111	54	39	42	<0.001
Rural	315	139	76	31	19	10	

^*^Ridit analysis: *u* = 6.1611 (urban = 0.5510; rural = 0.4468)

### Sociodemographic factors and dental care-seeking behavior

Several factors, including sex, education level, and income, were included in the logistic regression model assessing the frequency of dental care visits (within the past 2 years *vs* never or > 2 years ago). Univariate *χ*^2^ analysis showed that only residential location (i.e., urban or rural residence) was significantly associated with more recent dental care visits in both middle-aged (*χ*^*2*^ = 7.577, *P* < 0.01) and elderly (*χ*^*2*^ = 5.451, *P* < 0.01) participants (Table 5). On the basis of this analysis, residential location was entered as an independent variable in the multivariate logistic regression analysis. As shown in Table 6, residence was the only factor significantly associated with dental care-seeking behavior (*P* < 0.05 for both middle-aged and elderly groups).

**Table 5:** Sociodemographic factors and dental care-seeking behavior according to age group, n

Sociodemographic factor	Middle-aged			Elderly		
	*n*	Dental care visits within 2 years	Never having a dental care visit or visit > 2 years ago	*n*	Dental care visits within 2 years	Never having a dental care visit or visit > 2 years ago
Residence category						
Urban^a^	400	143	257	198	75	123
Rural	392	109	283	198	53	145
Sex						
Male	396	120	276	198	67	131
Female	396	132	264	198	61	137
Education level						
Illiteracy	48	16	32	100	22	78
Primary school	128	33	95	130	45	85
Middle school	299	95	204	95	32	63
High school or above	317	108	209	71	29	42
Income						
Low	581	167	414	335	104	231
High	211	77	134	61	24	37

^a^Univariate *χ* ^2^ analysis: middle-aged: *χ* ^*2*^ = 7.577; elderly: *χ* ^*2*^ = 5.451; both *P* < 0.01 compared to rural residence

**Table 6:** Logistic regression analysis of the association between residence categories and dental care-seeking behaviors

Variable	β^a^	SE	Wald	*df*	*P*	OR	95 % CI
Middle-aged							
Sex	0.166	0.158	1.104	1	0.293	1.181	0.866–1.610
Education level (1)	−0.294	0.397	0.549	1	0.459	0.745	0.342–1.623
Education level (2)	−0.134	0.369	0.133	1	0.716	0.874	0.424–1.803
Education level (3)	−0.215	0.380	0.319	1	0.572	0.807	0.383–1.700
Income	0.352	0.181	3.801	1	0.051	1.422	0.998–2.026
Residence^b^	−0.432	0.158	7.501	1	0.006	0.649	0.447–0.884-
Elderly							
Sex	−0.070	0.223	0.098	1	0.754	0.932	0.602–1.444
Education level (1)	0.585	0.308	3.612	1	0.057	1.795	0.982–3.281
Education level (2)	0.412	0.344	1.434	1	0.231	1.510	0.769–2.965
Education level (3)	0.629	0.384	2.687	1	0.101	1.876	0.884–3.982
Income	0.176	0.300	0.344	1	0.558	1.192	0.662–2.147
Residence^b^	−0.505	0.217	5.391	1	0.020	0.604	0.394–0.924

^a^Multivariable logistic regression analysis; ^b^comparing rural residence with urban residence

## Discussion

The current study shows that the frequency of dentist visits is low in both middle-aged and elderly populations in northeast China. The frequencies of middle-aged (82.3 %) and elderly (80.3 %) residents of northeast China who did not visit dentist in the past year were much higher than in comparable populations in south China (20.9–76.0 %) [25, 26] and in other developed countries (28.6–49.7 %) [23, 32, 33]. The high burden of oral disease and limited oral health care resources in China are preventing the dental care needs of elderly individuals from being adequately met [34], particularly in the central region of northeast China. Although northeast China, which bridges the Northeast Economic Zone and the Greater Bohai Economic Zone, is considered one of the key political, economic, and cultural centers of the nation, the data presented here indicate that both oral health status and oral health awareness in this region are low.

The perceived need for dentist visits in middle-aged populations was higher than in elderly populations, and higher in urban areas than in rural areas. Furthermore, the frequency of regular oral health checkup and periodontal treatment was higher in urban areas than in the rural areas. However, usage of oral health care service was generally low in both middle-aged and elderly populations in northeast China, and the rate of dental care visits was low. While 60–70 % of the subjects chose the option that “there is a need for dental care visit,” less than 20 % had visited a dentist during the past year. Less than 10 % of these chose periodontal treatment, while most of underwent tooth extraction or received fillings or inlays. These results reflect a substantial discrepancy between the needs and demands of oral health care service in middle-aged and elderly people in northeast China.

With regard to the reasons for dental care visits, due to a lack of basic oral health knowledge, we found that individuals had a generally high assessment of their oral health status compared to the mean level reported in previous studies [29, 30]. Approximately half of middle-aged and elderly people thought that there was no problem with their teeth, and there was therefore no need to visit a dentist. Moreover, less than 7 % of these individuals actively followed regular oral health checkup and took preventive measures, while more than 80 % of them visited dentists only when they had acute or chronic toothache. Furthermore, compared with residents of urban areas, more middle-aged people in rural areas reported a perceived absence of severe oral diseases or a lack of time as reasons not to visit dentists. Indeed, most middle-aged people in rural areas typically choose passive measures (self-medication, tolerance, etc.) because they consider their oral diseases to be not severe. In contrast, although the number of middle-aged people in urban areas who followed regular oral health checkup was similarly limited, these individuals reported visiting dentists in a relatively timely manner upon the onset of toothache symptoms. Reasons for these results, which are similar to those of previous studies [35–37], include differences in income level between rural and urban areas, and imbalances in the distribution of oral health care resources [38].

Consistent with the lack of coverage of rural areas by the traditional reimbursement system in China, and the fact that the new social health insurance system was in its early stages in rural areas during the study period, the majority of participants living in rural areas obtained dental care at their own expense. Whereas health insurance covered a certain proportion of dental care visits in urban areas, there were limits to such coverage and, which is likely why most participants living in urban areas also paid for their own dental care. Economic status is an important constraint on dental care-seeking behavior: 30–40 % of middle-aged and elderly participants could likely not afford dental care, even when it was definitely required. Accordingly, as they did not seek dental care services, the need for dental care is not reflected by demand, which compromises the use of the existing, limited oral health care resources [34, 39].

With regard to choices of dental care clinics, there was a significant difference between rural and urban areas in both the middle-aged and elderly groups. The results of this study show that most people who live in rural areas went to private clinics for their dental care. Our recent study [40] found a total of 1518 private clinics in central northeast China, accounting for 74.88 % of oral health resources. Most of these clinics are inexpensive, convenient, have no time limit for treatment, and are distributed in communities and villages and, as such, are emerging as the preferred choice for dental care in middle-aged and elderly individuals. These features are of great importance for people of limited financial means living in rural areas. However, the technical strength of such clinics is relatively weak; for example, only 33.2 % of their staff are registered as dentists, and only 42.5 % have a college degree [40], indicating a need for improved management and continuing education in these clinics [26]. The results of the present study also show that fewer people went to provincial comprehensive hospitals and dental specialized hospitals above the county level, particularly in rural areas. The relatively high expense could be an important factor restricting the participants from seeking dental care at such hospitals. In addition, these hospitals are generally located in city centers, and their service duration is limited, making it inconvenient for people who seek dental care service. The advantages of these hospitals include their advanced dental specialists with solid training and sophisticated treatment techniques. We suggest that such oral health resources should be better developed and utilized. For instance, these hospitals can allocate specialists and transfer technologies to community clinics, which will help bridge the gap between the needs and demands of dental care to improve oral health in northeast China.

Consistent with the findings of Lundegren et al. [12], the results of this study show that education levels are positively associated with tooth brushing frequency. Participants with higher educational levels reported brushing their teeth more often. Moreover, consistent with the data of Chaves et al. [41], the frequency of high income individuals who had never visited dentists was significantly lower than in lower income individuals. After educational level and income had been included in the logistic regression equation, however, there was no significant influence on the frequency of dental care visits. These data indicate that although education level and income influence the frequency of dental care visits, they cannot be considered as determinative factors of such visits. In contrast, education levels in central China are significantly correlated with oral health-related quality of life [28]. In addition to geographic disparities with other parts of the country, including differences in region and lifestyle, northeast China has, since the 1930s, been a prominent economic mega-region in China. In the years since the reformation and opening up and the accompanying changes in the economic system, the development of this region has gradually lagged behind the economically developed coastal regions. The restrictions that the lack of economic development in northeast China have placed upon lifestyle and culture in this region [42] have attenuated the overall impact of education level and income on dental care-seeking behavior.

Consistent with findings from previous studies [25, 32, 36, 37], the results presented here show that rural residence is a significant impediment to dental care-seeking behavior in middle-aged and elderly people. This phenomenon has a variety of explanations. First, as observed in this study, middle-aged and elderly people living in urban areas report greater access to oral health information than those living in rural areas. Greater access to oral health knowledge not only increases an individual’s awareness of oral health care, but can also improve the perception of a person’s need for dental care [24]. Second, health insurance coverage is higher in urban areas compared to rural areas. In rural areas, most of the middle-aged and the elderly participants obtained dental care at their own expense, suggesting that the low coverage of dental health insurance directly affects dental care-seeking behavior in this population. Because most rural residents have lower incomes than urban residents, they may not be able to afford such visits, which hampers the intent to seek dental care services. Finally, the availability of health services could also affect dental care-seeking behavior [37, 43]. In parallel with the rapid development of urban economies, the demand for oral health care is increasing among urban residents, and disparities in the allocation of oral health resources between urban and rural areas are increasing. Our previous study [44] showed that urban areas in central northeast China have eight times as many dentists as rural areas, and that the vast majority of oral health institutions and dental manpower services were located in cities. As a consequence, rural oral health services cannot meet the oral health needs of the majority of rural residents, which in turn limits active dental care-seeking behavior among middle-aged and elderly people in rural areas.

There are several limitations of the present study. First, information bias may be present, as all the data collected were self-reported by the participants, with no objective measures of oral health status provided by medical or experimental examination. Second, stratified random sampling was used to obtain the sample in the present study, thus members of the same family may have been included. Members from the same family are likely to share similar health behaviors, which could also introduce bias. Third, the degree of the variability in the urban areas may not be comparable with that in the rural areas; however, no data were available regarding these differences, which could bias the results. Finally, other sociodemographic factors, including smoking, weekly low alcohol consumption, body mass index > 25 mg/kg^2^, and fear of dental care, have been recently suggested as possible determinants of dental care-seeking behavior [10, 32]. Unfortunately, these data were not collected in the current study. We will address these limitations in future studies.

## Conclusion

The frequency of dental care visits and the utility of oral health resources are unsatisfactory among the middle-aged and elderly populations in northeast China, particularly in rural areas. Among sociodemographic factors, education level and income are positively associated with dental care visits, and rural residence is negatively associated with the frequency of such visits. We suggest that oral health knowledge be improved through oral health education and promotion, and that health insurance coverage for dental care, including preventive dental care, be expanded in rural areas.
